# The Treatment of Gastric Ulcer

**Published:** 1901-02-09

**Authors:** 


### THE TREATMENT OF GASTRIC ULCER.

Coming then to the question of treatment, Mr. Mayo Robson says that this is at first essentially medical, and that if properly carried out for a sufficient length of time it is usually completely successful. In many cases, however, either from the uncertainty of diagnosis or from the impatience of the subject, care in diet and rest are not persevered in for a sufficient length of time, and relapses result, until in the end complications supervene or the ulcer becomes chronic, and then in many cases surgical treatment is the only method capable of affording relief. Mr. Robson very rightly, we think, points out that relief to immediate symptoms does not by any means show that an ulcer of the stomach has healed, and he suggests that any case of gastric ulcer coming under observation should be treated rigidly for a much longer time than is at present considered necessary, and that on no account should the patient be allowed to get up or to resume solid diet until all pain and tenderness have disappeared for at least a fortnight. In regard to the operative treatment of these cases Mr. Mayo Robson has no doubt that if we could get at the statistics we should find that operations for the relief of gastric ulcer are being done with far greater frequency and with a far less mortality than was the case some time ago; and he fully believes that we shall shortly show a mortality of not more, and probably less, than 5 per cent. in all operations for simple as contrasted with malignant diseases of the stomach. In his own private work his mortality is under 5 per cent., and although not so many months ago he said that the surgical treatment of intractable gastric ulcer was still *sub judice*, he would now say that the surgical treatment of intractable or relapsing gastric ulcer is in the greater number of cases the only satisfactory method, and that operation should be resorted to at a much earlier period that has hitherto been the custom, and always before the patients are so far reduced by pain and starvation, or by the supervention of serious complications, that their weakened condition renders any operation a serious matter. He holds that ulcer of the stomach is a far more serious affair than the profession generally recognises, and that, considering the mortality of the cases medically treated, if surgery can save 95 per cent. of the more serious cases a real advance will have been made. The operation which of all others he relies upon most in cases of gastric ulcer is that of gastro-enterostomy, and he prefers the posterior operation, which he performs after a method of his own, making the junction between the stomach and the first part of the jejunum by means of two continuous sutures around a decalcified bone bobbin. The whole operation can, he says, be completed in half an hour, and he has done it in twenty minutes. His last twenty cases have all recovered without pain, vomiting, or any drawback.

